# Effects of Banhabaekchulcheonma-Tang on Brain Injury and Cognitive Function Impairment Caused by Bilateral Common Carotid Artery Stenosis in a Mouse Model

**DOI:** 10.7150/ijms.90167

**Published:** 2024-01-21

**Authors:** Da-Woon Kim, Jung-Hwa Lim, Suin Cho, Sang-Ho Kim

**Affiliations:** 1 Department of Neuropsychiatry of Korean Medicine, Pohang Korean Medicine Hospital, Daegu Haany University, 411 Saecheonnyeon-daero, Nam-gu, Pohang-si, Gyeongsangbuk-do, Republic of Korea.; 2 Department of Neuropsychiatry, School of Korean Medicine, Pusan National University, 49, Busandaehak-ro, Yangsan-si 50612, Republic of Korea.; 3 Pusan National University Korean Medicine Hospital, 20 Geumo-ro, Yangsan-si 50612, Republic of Korea.; 4 Department of Korean Medicine, School of Korean Medicine, Pusan National University, 49 Busandaehak-ro, Mulgeum-eup, Yangsan, Republic of Korea.

**Keywords:** vascular dementia, brain ischemia, bilateral carotid artery stenosis, banhabaekchulcheonma-tang, herbal medicine.

## Abstract

Vascular dementia (VD) is the second most prevalent dementia type, with no drugs approved for its treatment. Here, the effects of Banhabaekchulcheonma-Tang (BBCT) on ischemic brain injury and cognitive function impairment were investigated in a bilateral carotid artery stenosis (BCAS) mouse model. Mice were divided into sham-operated, BCAS control, L-BBCT (40 ml/kg), and H-BBCT (80 ml/kg) groups. BBCT's effects were characterized using the Y-maze test, novel object recognition test (NORT), immunofluorescence staining, RNA sequencing, and Kyoto Encyclopedia of Genes and Genomes (KEGG) pathway and Gene Ontology (GO) analyses. The NORT revealed cognitive function improvement in the H-BBCT group, while the Y-maze test revealed no significant difference among the four groups. The CD68+ microglia and GFAP+ astrocyte numbers were reduced in the H-BBCT group. Furthermore, H-BBCT treatment restored the dysregulation of gene expression caused by BCAS. The major BBCT targets were predicted to be cell division cycle protein 20 (CDC20), Epidermal growth factor (EGF), and tumor necrosis factor receptor-associated factor 1 (TRAF1). BBCT regulates the neuroactive ligand-receptor interaction and neuropeptide signaling pathways, as predicted by KEGG and GO analyses, respectively. BBCT significantly improved cognitive impairment in a BCAS mouse model by inhibiting microglial and astrocyte activation and regulating the expression of CDC20, EGF, TRAF1, and key proteins in the neuroactive ligand-receptor interaction and neuropeptide signaling pathways.

## Introduction

Dementia is a neurodegenerative disease characterized by memory loss and cognitive impairment. The global prevalence and economic burden of dementia is increasing rapidly. It has been estimated that the number of people with dementia will increase from 57.4 million cases globally in 2019 to 152.8 million cases in 2050 1. In South Korea, the total annual cost of dementia in 2020 was approximately 13 billion US dollars, which is estimated to increase to approximately 43 billion US dollars by 2040 2. Vascular dementia (VD), the second most prevalent type of dementia after Alzheimer's disease, accounts for 15% of all dementia cases and is caused by ischemic or hemorrhagic brain tissue damage 3. Cerebrovascular diseases such as arteriolosclerosis, cortical and subcortical microinfarction, cerebral amyloid angiopathy, and white matter degeneration, which involves myelin loss and axonal abnormalities, contribute to vascular dementia 4.

Currently, no medication has been proven to be sufficiently effective against VD. Memantine and acetylcholinesterase inhibitors (AChEIs), including donepezil, galantamine, and rivastigmine, are commonly used to treat cognitive impairment in VD 5. However, a recent meta-analysis revealed that these medications had only minor effects in patients with VD 5. Furthermore, AChEIs are associated with adverse events including vomiting, diarrhea, headache, and hypertension 5. Due to the long-term administration of anti-dementia drugs, research on more effective and safe drugs is needed. Recently, multitarget drugs have attracted considerable interest for the treatment of dementia, and traditional herbal medicines, consisting of various herbs, could also have multiple targets. Previous study has characterized the mechanism of the complex anti-dementia effect of traditional herbal medicine 6.

Banhabaekchulcheonma-Tang (Banxia-Baizhu-Tianma-Tang, BBCT) is commonly used for VD with a pattern of phlegm turbidity obstructing the orifices in South Korea. BBCT comprises Pinelliae Tuber, Hordei Fructus Germinatus, Citri Unshius Pericarpium, Atractylodis Rhizoma, Atractylodis Rhizoma Alba, Massa Medicata Fermentata, Ginseng Radix, Astragali Radix, Poria Sclerotium, Gastrodiae Rhizoma, Alismatis Rhizoma, Zingiberis Rhizoma, Phellodendri Cortex, and Zingiberis Rhizoma Recens 7. Previous studies have reported the anti-oxidant, anti-inflammatory, and antihypertensive effects of BBCT 8-10. In clinical studies, BBCT was found to improve the activities of daily living in patients with cerebral infarction 11, and cognitive function in patients with VD 12. A study on a rat model of middle cerebral artery occlusion (MCAO) revealed that BBCT had neuroprotective effects on hippocampal neurons and increased acetylcholinesterase expression 13.

Among the VD rodent models, the 2-vessel occlusion (bilateral common carotid artery occlusion) model is mainly used in rats. However, the underdevelopment of the posterior communicating arteries of the circle of Willis causes severe ischemia during 2-vessel occlusion in mice 14. Furthermore, the unilateral carotid artery occlusion model cannot easily induce VD because of the sufficient blood supply through the other carotid artery. However, BCAS using micro-coils wrapped around both common carotid arteries can induce chronic cerebral hypoperfusion, cerebral inflammation, white matter lesions, gliosis, decreased blood-brain barrier integrity, and working memory deficits 15,16. Thus, it has been proposed to be the most appropriate model for VD 15. However, no previous study has used the BCAS model to observe the anti-dementia effects and mechanisms of BBCT in VD. Therefore, in this study, we used the BCAS mouse model to investigate the anti-dementia and neuroprotective effects of BBCT against ischemic brain damage.

## Materials and Methods

### Animals

Male C57BL/6 mice, 6-week-old (Samtako, Osan, Korea) weighing 20-22 g, were housed in polypropylene cages at 24 ± 4 °C under 12 h light and dark cycle for 1 week or more prior to the experiment, with access to sufficient solid feed and water. The animal experimental protocol was approved by the Institutional Animal Care and Use Committee of the Pusan National University (approval number: PNU-2021-0260).

### Preparation of BBCT

Overall, 14 species of dried medicinal herbs used in this study were supplied by Pohang Korean Medicine Hospital of Daegu Haany University (Pohang, Korea). The detailed composition of BBCT is provided in Table 1 7.

In clinical practice, the herbs in quantities presented in Table 1 are boiled in water and reduced to 360 ml, and adults weighing 60 kg are administered the whole decoction, 120 ml at a time, thrice a day. In this study, the mixture of medicinal herbs (four times the amount presented in Table 1) was boiled for 2 h in 2400 ml of distilled water using an herbal solution heater (Dae Woong, Seoul, Korea) to obtain a final volume of 1440 ml. When boiling the herbs, non-woven fabric was used to filter out large debris, and the debris that was not filtered out was removed by centrifuging the herbal solution at 16,000 ×g (MICRO 17TR, Hanil Science, Daejeon, Korea). The dosage in humans is 360 ml/60 kg (6.0 ml/kg), and the metabolic rate of mice is approximately 13 times that of humans; therefore, the dosage in mice was calculated to be 80 ml/kg 17. Two different concentrations (80 ml/kg and 40 ml/kg) were administered to two sets of mice for comparison. The decoction was administered orally to both groups. In the 40 ml/kg group, the decoction was diluted 2-fold to maintain the same volume.

### BCAS procedure

BCAS was used to induce VD. Mice were anesthetized with N_2_O/O_2_ (70%/30%, Woojingastech, Gimhae, Korea) and isoflurane (Troikaa Pharmaceuticals, Ltd., Gujarat, India). A midline incision was made in the neck region and both common carotid arteries were carefully separated from the surrounding tissues. The arteries were then wrapped with a micro-coil (0.18 mm inner diameter, Sawane Co., Shizuoka, Japan) to induce stenosis. The sham-operated group underwent the same surgical procedure without BCAS.

### Classification of experimental groups and administration of BBCT

**Figure 1:**
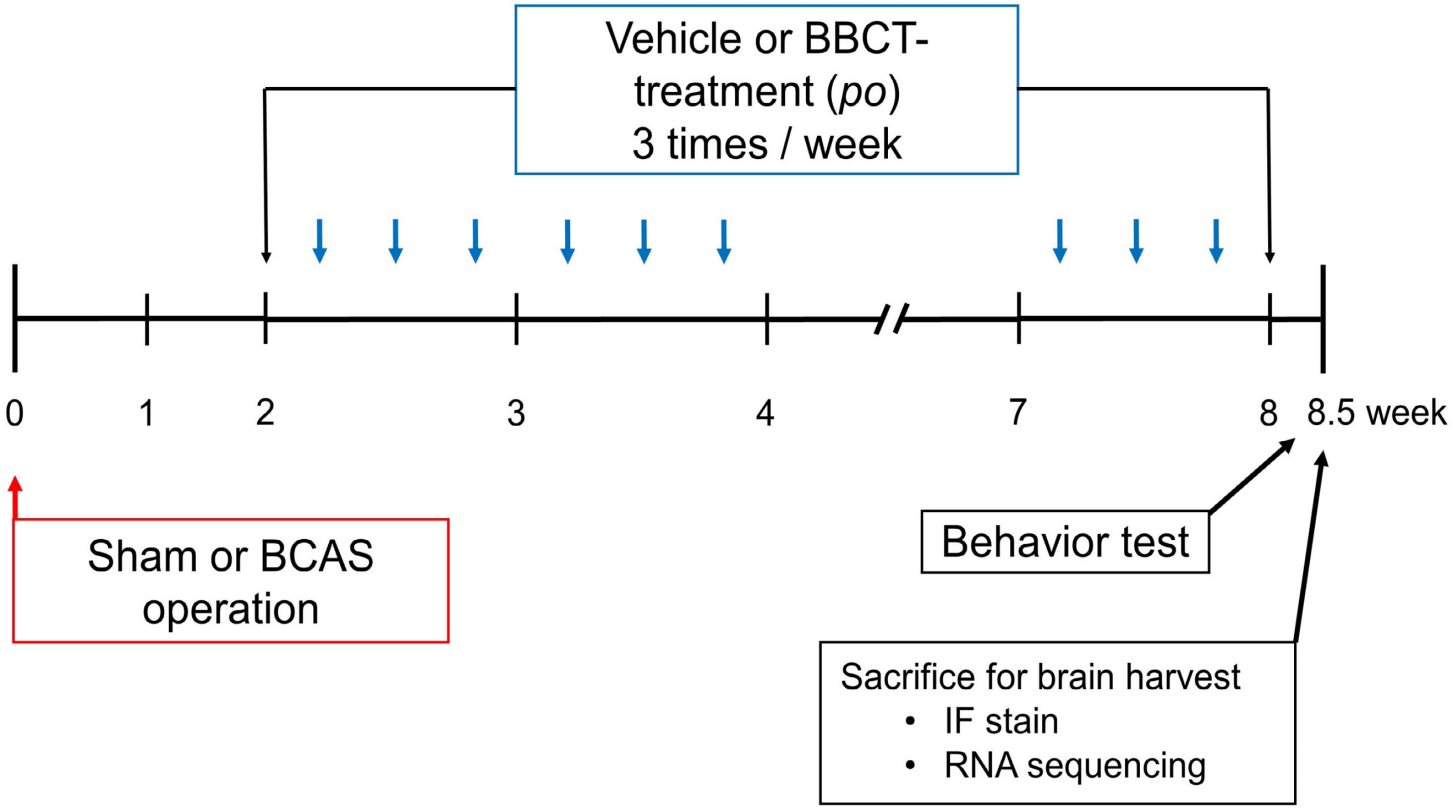
Schematic representation of the experimental procedure. Vehicle (80 ml/kg) was administered to the sham-operated and BCAS control groups and BBCT to the L-BBCT (40 ml/kg) and H-BBCT (80 ml/kg) groups three times a week for 6 weeks after 2 weeks of surgery. Body weight changes, blood pressure, and heart rate were measured once every 2 weeks. Behavioral tests, including the Y-maze test and novel object recognition test, were performed 8 weeks after surgery, and mice were sacrificed for immunofluorescence staining (IF staining) and RNA sequencing.

In this study, mice were divided into four groups: (1) sham-operated group, (2) BCAS control group, (3) low concentration BBCT (L-BBCT) group, and (4) high concentration BBCT (H-BBCT) group. Each group consisted of five mice. The sham-operated and BCAS control groups were orally administered equal volumes of vehicle (80 ml/kg). The L-BBCT and H-BBCT groups were administered 40 and 80 ml/kg BBCT, respectively. Vehicle and BBCT were administered three times a week for 6 weeks, starting at 2 weeks after surgery. The experimental procedure is illustrated in Figure 1.

### Y-maze test

Working memory was tested using a Y-shaped maze consisting of three arms (35 cm long, 40 cm high, and 7 cm wide). During the 2-min habituation period, one arm was closed, and each mouse was placed at the center of the maze and allowed to explore the two open arms. After opening the closed arm, each mouse was allowed to freely explore the three arms for 8 min. A digital video camera (HDR-CX450; Sony Co., Tokyo, Japan) was used to record the behavior of each mouse. Spontaneous alternation was reported when a mouse entered the three arms sequentially (C-A-B, B-C-A, and A-B-C; Figure S1). The following equation was used to assess the spontaneous alternation behavior:

% Alternation triplet = ((number of alternations) / (total number of arm entries - 2)) × 100

### Novel object recognition test

The novel object recognition test (NORT) assesses recognition memory in rodents using their natural tendency to explore novelty. During the habituation period, each mouse was given 10 min to explore the open-field box (gray box, 40 × 40 × 40 cm (height)) without any objects. In the acquisition session, two identical objects were placed in opposite corners of the field box (Zones 2 and 3; Figure S2), allowing each mouse to explore both objects for 10 min. After 20 min and replacing the original object (familiar, F) in Zone 3 with a new object (new, N), the mouse was placed back into the field box and allowed to explore the objects for 10 min (recognition session). The two objects and field box were cleaned within each session using 70% ethanol. A digital video camera was used to record the time spent in exploring each object. The discrimination ratio was calculated using the following equation:

% discrimination ratio = (total N time) / (N time + F time) × 100

### Body weight and physiological parameters measurement

The body weights of the mice were measured once every 2 weeks. Blood pressure and pulse rate were measured from the tail of the mice using a non-invasive blood pressure analyzer (BP-2000, Visitech System, North Carolina, USA) once every 2 weeks. At the end of the experiment, blood samples—collected under deep anesthesia—were centrifuged at 1,500 ×*g* for 15 min at 4 °C (MICRO 17TR, Hanil Science, Daejeon, Korea) to obtain serum. Serum electrolytes, including sodium (Na+), potassium (K+), and chloride (Cl-), were measured using an electrolyte analyzer (Dri-Chem 3500i, Fuji, Tokyo, Japan) to eliminate the possibility of an electrolyte imbalance.

### Cryosection of mice brain

Transcardiac perfusion with phosphate-buffered saline (PBS; Bio Basic, Ontario, Canada), followed by 4% paraformaldehyde, was performed. The mice brains were then fixed in 4% paraformaldehyde and 10-30% sucrose at 4 °C for 3 days. The mice brains were then placed in 10%, 20%, and 30% sucrose in order, then frozen in optimal cutting temperature compound (Thermo Fisher Scientific, Massachusetts, USA), and stored at -80 °C. Thick brain sections (25 μm) were collected using a cryostat (Leica, Wetzlar, Germany). The sections were placed on glass slides and stored at -80 °C.

### Immunofluorescence staining of mice brain tissue

The brain sections were incubated with 5% bovine serum albumin (A3294-10G, Sigma-Aldrich, Missouri, USA) for 1 h at 25 °C. After washing the blocking buffer, the sections were incubated with primary antibodies including anti-CD68, anti-NeuN, and anti-GFAP antibodies (#97778, #94403, #12389, Cell Signaling Technology, Massachusetts, USA, respectively), at 4 °C for 12 h. Then, the sections were washed in PBS and incubated with secondary antibodies (goat anti-mouse IgG H&L Alexa 488, Alexa 555 or goat anti-rabbit IgG H&L Alexa 488, Alexa 555, Abcam, Cambridge, UK) for 2 h at 25 °C. Finally, the sections were washed in PBS and mounted on slides using the Fluoroshield mounting medium with DAPI (ab104139, Abcam). Fluorescence images of the brain tissue were obtained using a fluorescence microscope (Ni-U, Nikon, Tokyo, Japan), and tissue images were merged using ImageJ software (NIH, Maryland, USA).

### RNA sequencing analysis

After the behavioral tests, the mice were sacrificed using a CO_2_ chamber. The mouse brain cortex (including white matter) was isolated and rapidly frozen. Total RNA was extracted using TRIzol (T9424-25ML, Sigma-Aldrich, Missouri, USA), and RNA quality was measured by capillary electrophoresis (2100 Bioanalyzer, Agilent, California, USA). mRNA was isolated from total RNA, cDNA was synthesized from the mRNA to prepare a cDNA library, and the prepared libraries were sequenced. RNA from sham-operated mice was used as a reference, and a change in expression from the baseline 2-fold was considered up- or down-regulated. Relative changes in expression were analyzed and visualized as a heat map using a multiple experiment viewer (MeV, https://mev.tm4.org). A protein-protein interaction (PPI) network was constructed using the Search Tool for the Retrieval of Interacting Genes/Proteins (STRING database, ver. 11.5). Kyoto Encyclopedia of Genes and Genomes (KEGG) pathway and Gene Ontology (GO) enrichment analyses, including biological processes, cellular components, and molecular function terms, were used to identify possible biological mechanisms.

### Statistical analysis

Statistical analyses were conducted using SigmaPlot software (version 12.0; Systat Software Inc., CA, USA). Data are expressed as mean ± standard deviation (SD) and were analyzed by one-way analysis of variance (ANOVA) followed by the Holm-Sidak test if data were normally distributed or by the Tukey test if the data were non-normally distributed. A *P*-value of < 0.05 was used to define statistical significance.

## Results

### Effects of BCAS operation and BBCT on body weight and physiological parameters in mice

**Figure 2:**
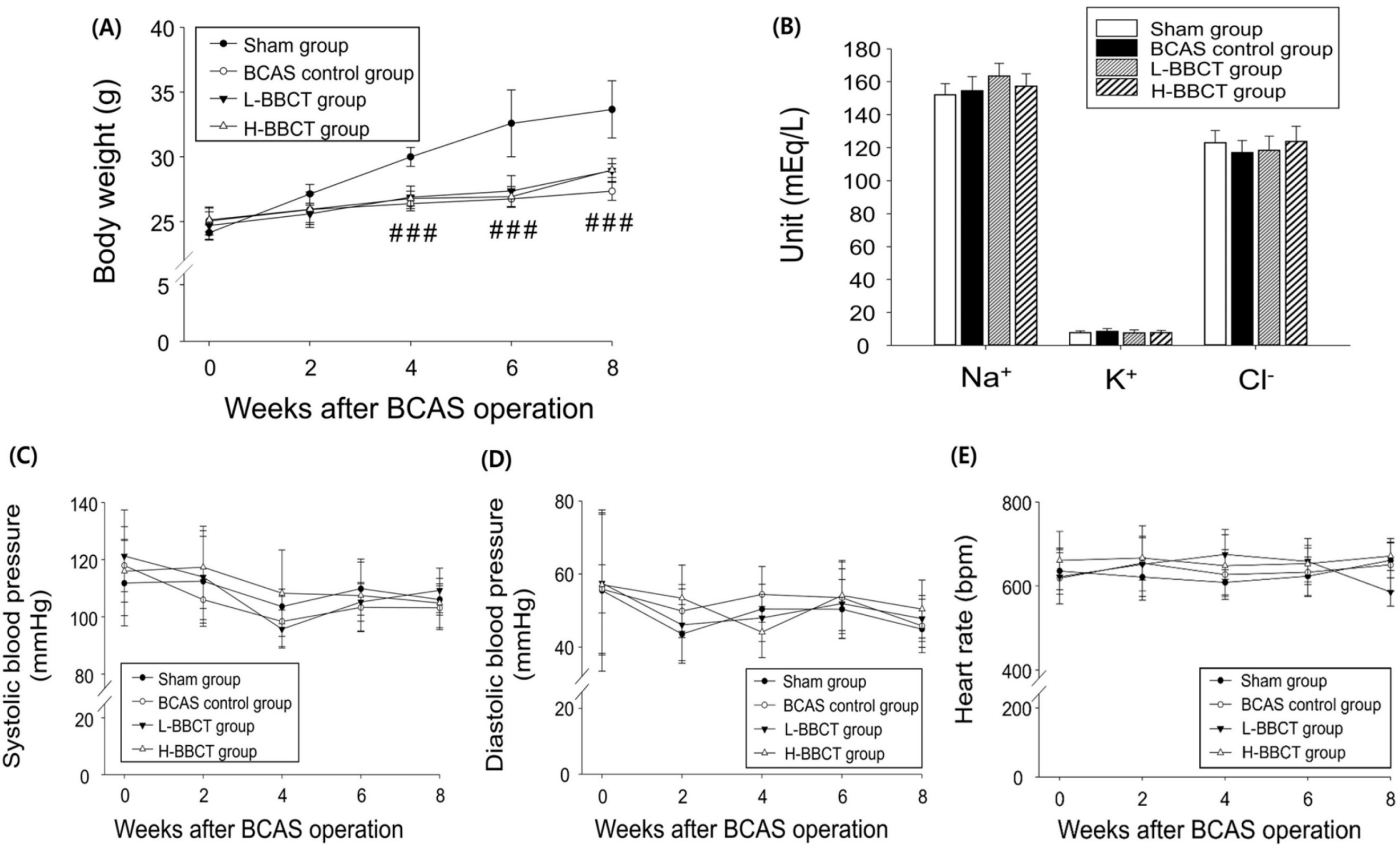
Influence of BCAS operation and effects of BBCT on body weight (A), serum electrolytes (B), systolic blood pressure (C), diastolic blood pressure (D), and heart rate (E) during 8 weeks in mice. The mice's body weight, blood pressure, and heart rate were measured once every 2 weeks. At the end of the experiment, the concentrations of Na^+^, K^+^, and Cl^-^ in serum samples were measured. Results are presented as mean ± SD.

After BCAS, a significant decrease in body weight was observed from week 4, compared to that in the sham-operated group. However, no significant difference was observed in body weight between the L-BBCT, H-BBCT, and BCAS control groups. None of the groups exhibited significant differences in blood pressure, heart rate, and serum electrolyte levels (Figure 2).

### Effects of BBCT on BCAS-induced cognitive function impairment in mice

**Figure 3:**
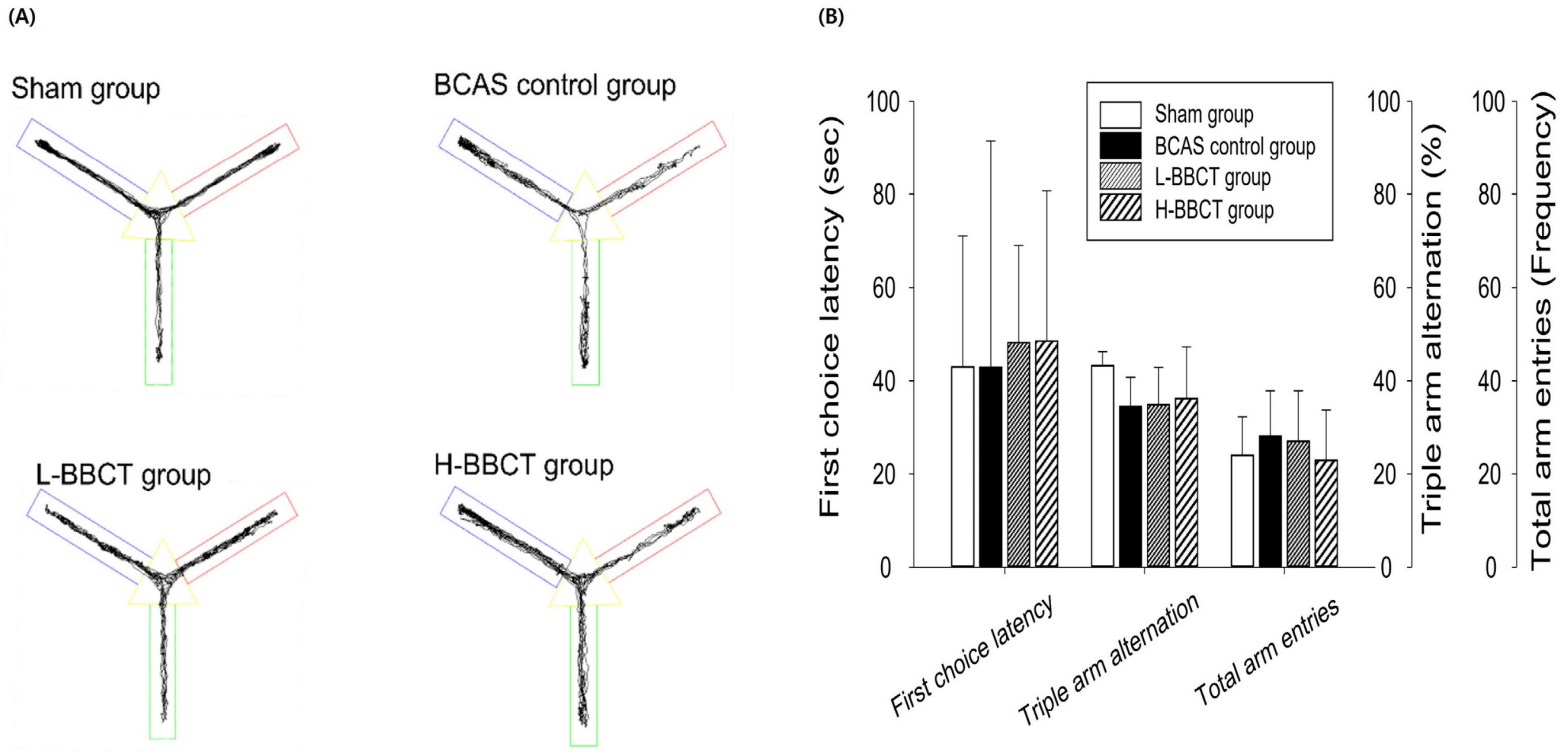
Top view of the Y-maze and the movement traces (A), latency to first-arm entry, the total number of entries into the three arms, and spontaneous alternation rates (B). All four groups had no significant difference in first-choice latency, total arm entries, and triple arm alternation ratio. Results are presented as mean ± SD.

In the Y-maze test, no significant difference was observed in the latency to first-arm entry, total number of entries into the three arms, and spontaneous alternation rates among the four groups (Figure 3).

**Figure 4:**
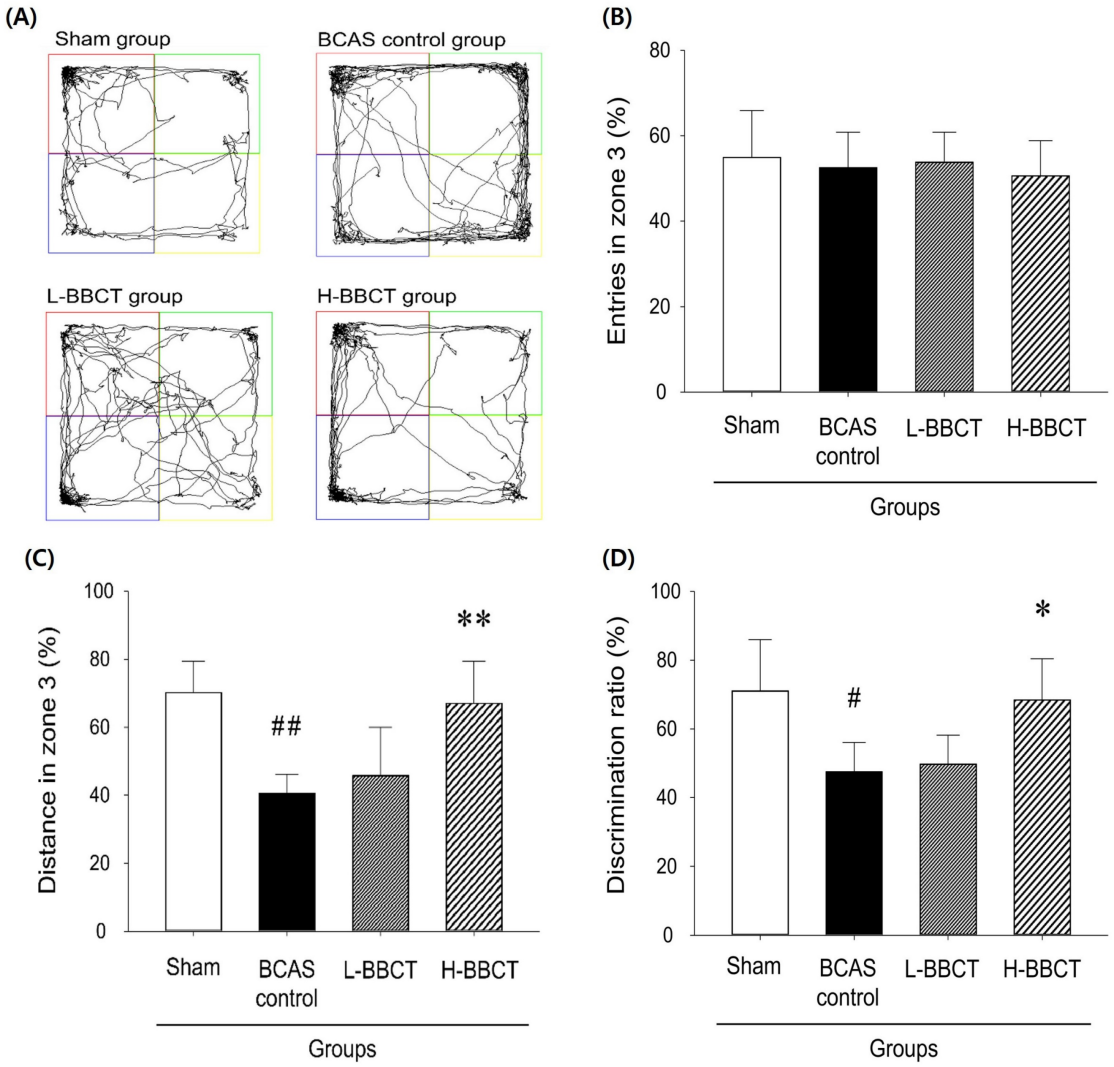
Top view of the square chamber for NORT and the movement traces (A), the entries in zone 3 in percentages (%) (B), the distance traveled in zone 3 in % (C), and the discrimination ratio (D). A new object was placed in the lower left corner of the square (zone 3), while an original object was placed in the upper-right corner of the square (zone 2). The distance traveled in zone 3 and the discrimination ratio were significantly decreased in the BCAS control group compared to the sham-operated group but increased significantly in the H-BBCT group compared to the BCAS control group. Results are presented as mean ± SD.

During the NORT, the distance traveled in zone 3, number of entries into zone 3, and time spent in zones 2 and 3 were analyzed. Figure 4A presents the movement traces of the mice. Zone 3 is the lower-left corner of the square (blue) where the original object was replaced with a new object, and zone 2 is the upper-right corner (green) where the original object was not replaced. Figure 4B reveals that no significant difference was observed in the entries into zone 3 (%) among the four groups. However, the distance traveled in zone 3 (%) and the discrimination ratio (%) were significantly lower in the BCAS control group, and the administration of H-BBCT attenuated this change (Figure 4).

### Effects of BBCT on microglia and astrocytes activation in BCAS model mice brains

**Figure 5:**
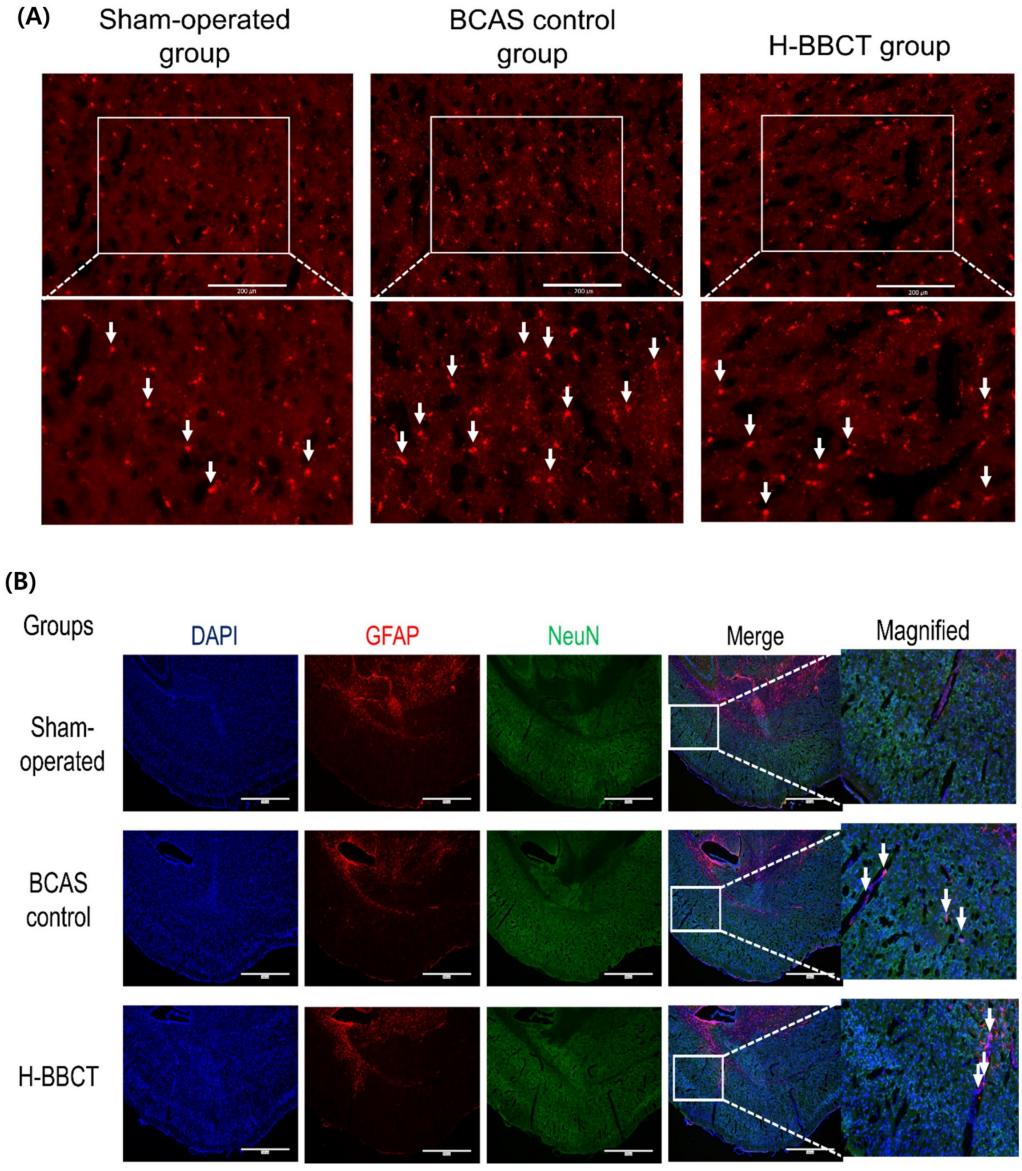
H-BBCT attenuated the BCAS-induced activation of microglia and astrocytes in mice brain cortex. (A) Representative images of IF staining of CD68 in mice brain cortex. Arrows: CD68+ microglia. Scale bar = 200 μm. (B) Representative images of IF staining of GFAP in mice brain cortex. The merged images present combinations of three images (DAPI, GFAP, NeuN). Arrows: GFAP+ astrocytes. Scale bar = 1000 μm.

We observed the expressions of CD68 and GFAP in the mouse cortex using immunofluorescence staining. The number of CD68+ microglia increased after BCAS but decreased after H-BBCT administration (Figure 5A). Additionally, Figure 5B reveals that GFAP+ astrocytes increased in the brains of BCAS control mice while they decreased in the brains of H-BBCT mice.

### Effects of BBCT on gene expression

Gene expression patterns in the cortex of mice were analyzed. In total, 386 genes were hierarchically clustered based on ≥2-fold variations in expression between the BCAS control and sham-operated groups (Figure S3). Figure S3C-D presents genes up-regulated and down-regulated in the BCAS control group (B) compared to the sham-operated group (S). H-BBCT administration restored the expression of these genes (H). The detailed alterations and restoration of gene expression are presented in Figure S4. Among the 223 genes up-regulation in the BCAS group, 178 (79.8%) were restored by H-BBCT (Figure S4C). Among the 163 down-regulated genes, 125 (76.7%) were restored by H-BBCT (Figure S4D).

**Figure 6:**
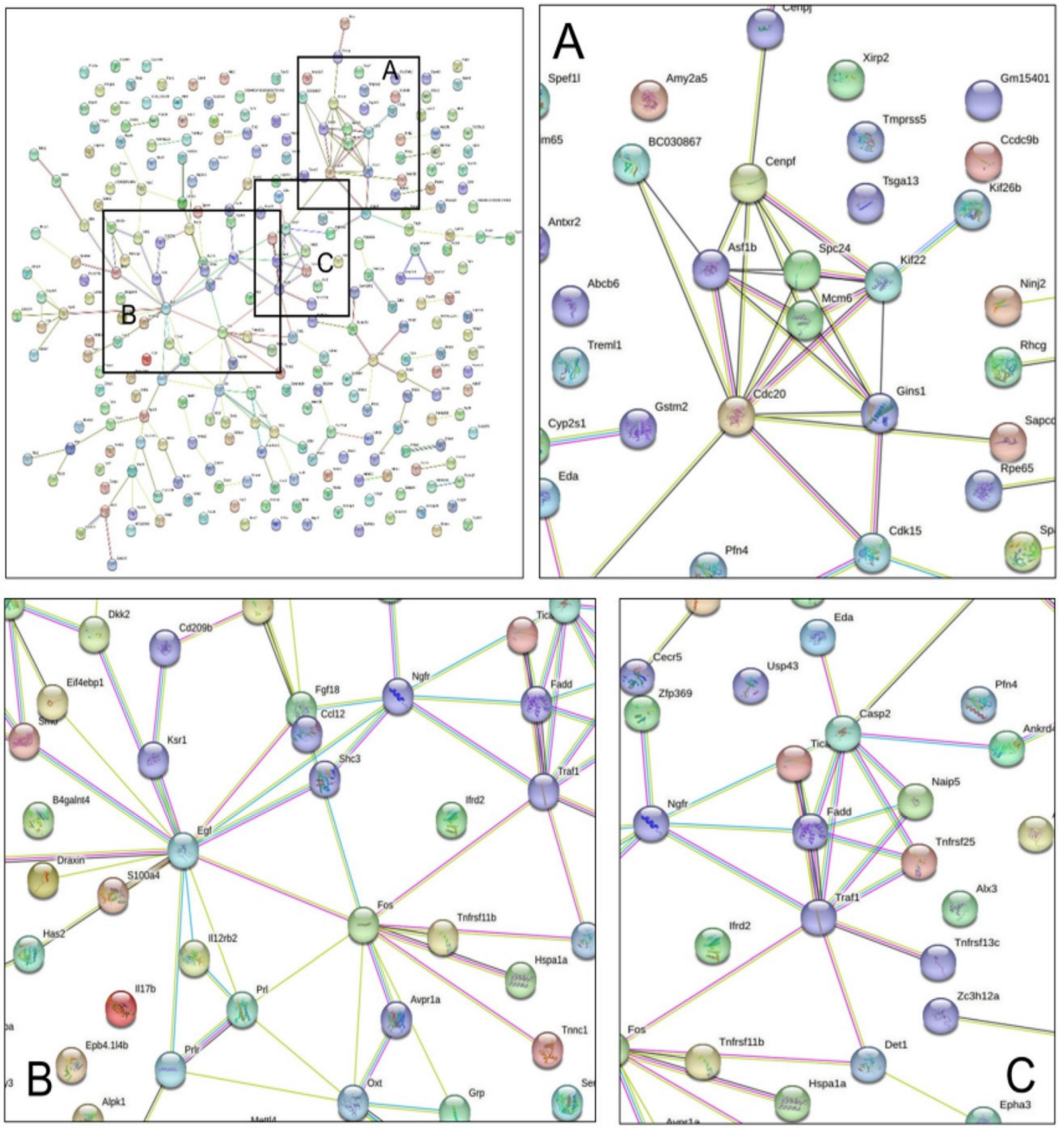
Protein network analysis using STRING database. Data on the restoration of gene expression after H-BBCT administration in BCAS model mice were uploaded to the STRING database to identify interactions between related proteins. The nodes indicate proteins, and the edges indicate protein-protein interactions. Three main protein-protein interactions were identified, and CDC20 (A), EGF (B), and TRAF1 (C) were expected to play key roles in these interactions.

We constructed a PPI network and identified the main target proteins expected to play key roles using the STRING database with 303 genes whose expression was restored by H-BBCT administration (Figure 6).

### GO and KEGG pathways enrichment analysis

**Figure 7:**
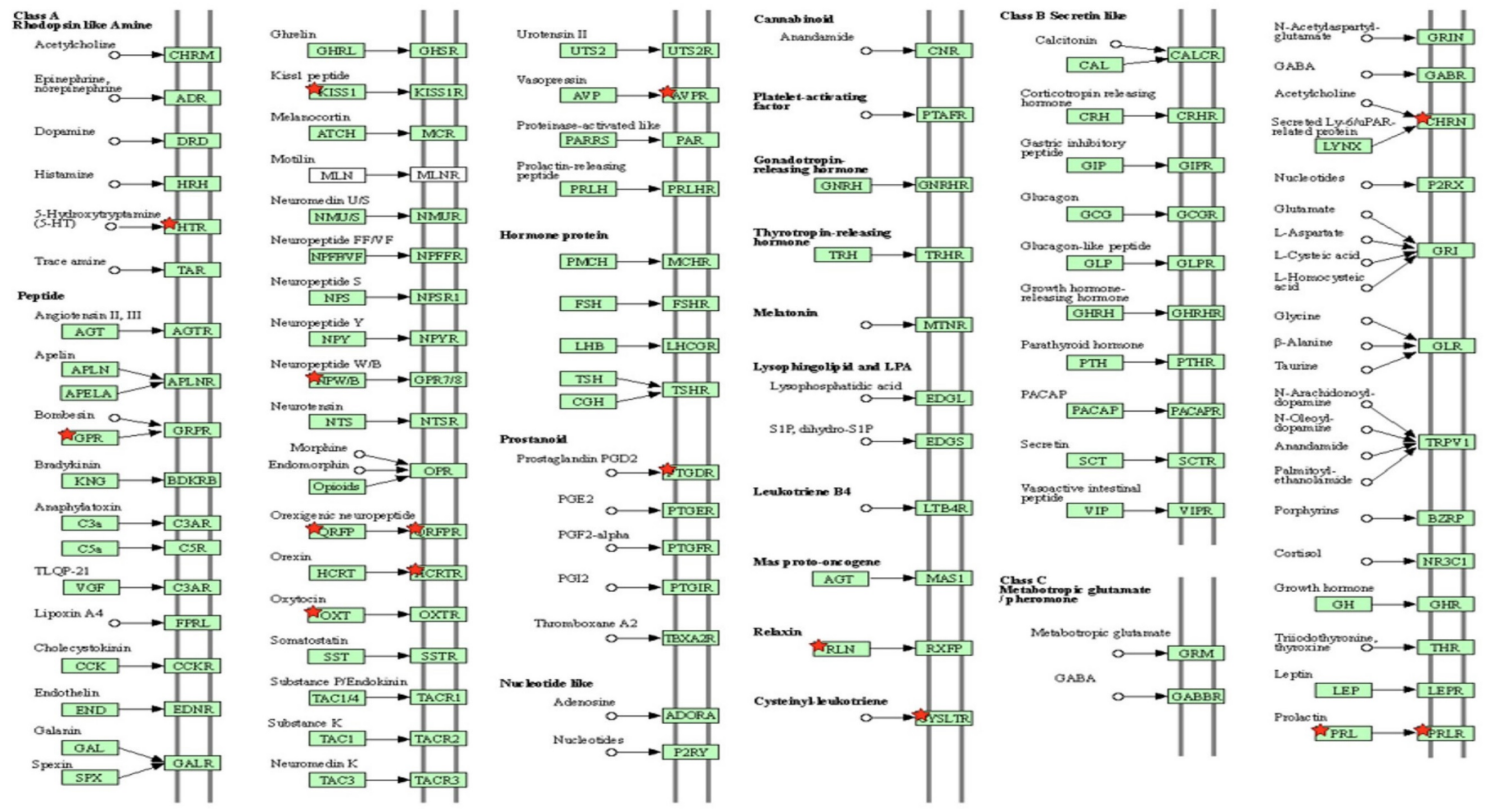
KEGG pathway map of neuroactive ligand-receptor interaction. The key targets of BBCT are marked with a red star. HTR: 5-hydroxytryptamine receptor; GPR: gastrin releasing peptide; KISS1: Kiss-1 metastasis suppressor; NPW/B: neuropeptide W/B; QRFP: pyroglutamilated RFamide peptide; QRFPR: QRFP receptor; HCRTR: hypocretin receptor; OXT: oxytocin-neurophysin 1; AVPR: arginine vasopressin receptor; PTGDR: prostaglandin D2 receptor; RLN: relaxin; CYSLTR: cysteinyl leukotriene receptor; CHRN: nicotinic acetylcholine receptor; PRL: prolactin; PRLR: prolactin receptor.

A KEGG pathway analysis of the genes restored by H-BBCT was performed. Nine main pathways, including neuroactive ligand-receptor interaction, cytokine-cytokine receptor interaction, and prolactin and interleukin-17 (IL-17) signaling pathways, were identified (*P* < 0.05, Table 2). Among these pathways, the neuroactive ligand-receptor interaction, involved in most genes, is presented in Figure 7.

GO enrichment analysis of the genes restored by H-BBCT revealed that the restored genes were enriched in biological processes including the neuropeptide signaling pathway (Table S1), cellular components such as the extracellular region (Table S2), and molecular functions such as RNA methyltransferase activity (Table S3).

## Discussion

In this study, we investigated the effects of BBCT on VD using a BCAS mouse model. BBCT administration improved cognitive impairment induced by chronic cerebral hypoperfusion and reduced the activation of microglia and astrocytes in the brain cortex. To observe gene expression changes and identify differentially expressed genes, we performed RNA sequencing on the H-BBCT group, which exhibited a significant cognitive improvement compared to that in the BCAS control group. Genes restored by BBCT administration were investigated using the STRING database, KEGG pathway analysis, and GO enrichment analysis.

In a previous clinical study Hao 12, BBCT combined with moxibustion significantly improved cognitive function than did nimodipine alone in patients with VD. Previous studies have characterized the effect of BBCT extensively. BBCT relaxes phenylephrine-induced arterial contractions and exerts endothelial protective effects by reducing reactive oxygen species (ROS) and high-sensitivity C-reactive protein levels in human umbilical vein endothelial cells 8,9. Moreover, BBCT has been reported to possess antihypertensive, anti-oxidant, and cardioprotective effects by reducing IL-1, IL-6, inducible nitric oxide synthase, and tumor necrosis factor-α (TNF-α) levels and suppressing the activity of the nuclear factor kappa light chain enhancer of activated B cells (NF-κB) pathway in hypertensive rats 10,18. In MCAO-induced VD model rats, BBCT improved cognitive dysfunction in an 8-arm radial maze task, exhibited neuroprotective effects, and increased acetylcholinesterase in the hippocampus 13. Among the components of BBCT, gastrodin of Gastrodiae Rhizoma attenuated amyloid-β (Aβ) deposition and glial activation in the brain of Alzheimer's disease model mice and inhibited abnormal phosphorylation of Aβ and Tau in the hippocampus of VD model rats 19,20. Furthermore, Astragalus polysaccharide suppressed apoptosis and Aβ accumulation in an Alzheimer's disease mouse model via the nuclear factor erythroid-2-related factor 2 (Nrf2) pathway, and Atractylodes macrocephala degraded amyloid precursor protein by regulating lysophagy *in vitro* 21,22.

NORT revealed that BCAS resulted in a decreased discrimination ratio, representing recognition memory impairment, consistent with findings of previous studies that used BCAS model mice 23,24. Moreover, H-BBCT administration was observed to improve recognition memory impairment in mice after BCAS. However, we observed no significant differences in the latency to first-arm entry, total number of entries into the three arms, and spontaneous alternation rates among all groups in the Y-maze test. The spontaneous alternation rate, which represents spatial working memory, decreased in the BCAS control group compared to that in the sham-operated group; however, the difference was insignificant. BCAS and administration of BBCT did not affect the working memory of mice based on the Y-maze test. In previous studies that used the BCAS model, working memory deficits were observed in the Y-maze test after BCAS 24,25. This difference in results may be due to differences in the surgical procedure and the degree of brain damage caused by ischemia. Additionally, in a study 13 using MCAO model rats, BBCT administration improved working memory deficits in an 8-arm radial maze. The types of rodents, cerebral ischemia models, and mazes used to evaluate working memory function may have contributed to this difference.

Microglia and astrocytes are neuroglial cells that contribute to post-ischemic inflammatory responses in the brain. CD68 and GFAP are common markers of microglia and astrocytes, respectively. Activated microglia secrete inflammatory cytokines and oxidative metabolites, such as TNF-α, IL-6, IL-1β, NO, and ROS contributing to neuronal cell damage 26. Cerebral ischemia induces astrocytes to release inflammatory factors, such as IL-1, IL-6, and TNF-α, and neurotoxins that may kill matured oligodendrocytes or inhibit the maturation of oligodendrocyte precursor cells 27. This reactive gliosis induced by chronic cerebral hypoperfusion contributes to axonal degeneration, demyelination, loss of white matter integrity, and cognitive impairment in mice 25,28,29. Consistent with previous reports, we observed increased activation of microglia and astrocytes after BCAS, using immunofluorescence staining of the mouse cortex 16,30, and the administration of H-BBCT attenuated this glial activation.

Based on the genes regulated to a normal level after administration of H-BBCT, we constructed a PPI network using the STRING database and identified important target proteins such as cell division cycle protein 20 (CDC20), epidermal growth factor (EGF), and tumor necrosis factor receptor-associated factor 1 (TRAF1). CDC20 is an essential cell cycle regulator for organisms to complete mitosis. CDC20 is an essential cell cycle regulator for organisms to complete mitosis. CDC20 activates the anaphase-promoting complex (APC) during cell division, and CDC20-APC regulates dendrite growth and presynaptic differentiation of neurons 31. EGF exhibits neuroprotective effects against ischemic injury and prevents cerebrovascular damage 32. Furthermore, EGF regulates oligodendrocyte progenitor cells, relevant to the remyelination capacity degradation in Alzheimer's disease or brain aging 33. TRAF1 plays an important role in apoptosis and inflammatory responses in cerebral ischemia-reperfusion injuries 34. Especially, the expression of TRAF1 increased after cerebral ischemia in mice, promoting neuronal apoptosis and exacerbating ischemic brain injury 34. Our results revealed that BBCT may prevent cerebral ischemic neuronal injury by regulating the expression of CDC20, EGF, and TRAF1.

KEGG pathway analysis of the restored genes revealed that the BBCT was mainly involved in the neuroactive ligand-receptor interaction pathway. This pathway consists of receptors and ligands responsible for signal transduction in the plasma membrane, and the expression of these receptors is associated with cognitive functions, including learning and memory 35-37. HTR (5-hydroxytryptamine receptor, a 5-HT receptor) regulates the activity of cerebral glutamatergic and cholinergic neurons in learning and memory 38. Several studies have suggested that activation of the 5-HT_1A_ receptor could prevent cerebral ischemia-induced neuronal injury in rodents 39,40. Notably, increased 5-HT_1A_ receptor binding in the temporal cortex of patients with VD was associated with preserved global cognitive function and memory 41. Gastrin-releasing peptide (GRP) is widely distributed throughout the brain, including the hippocampus, and regulates cell growth and proliferation during development, emotional responses, and memory in the central nervous system 42. GRP modulates glutamatergic transmission in the hippocampus to improve cognitive function and synaptic plasticity in VD model rats 43. Cysteinyl leukotriene receptor (CYSLTR) modulates overall neuroinflammatory and repair processes of cerebral ischemia, such as microglial inflammation, blood-brain barrier leakage, and glial scar formation, and its antagonists could attenuate neuronal damage and activation of microglia and astrocytes in rats 44,45. CHRN (Nicotinic acetylcholine receptor, nAChR) agonists have been developed for Alzheimer's disease 46. The α7 nAChR subtype mediates the brain cholinergic anti-inflammatory pathway, resulting in the negative regulation of the pro-inflammatory cytokines 47. Furthermore, the activation of α7 nAChR in mouse microglia and astrocytes exhibits anti-inflammatory effects 48,49. The mechanism underlying the reduced activation of glial cells and improved cognitive function after BBCT administration may be the regulation of these receptors or ligands by BBCT in the neuroactive ligand-receptor interaction pathway. GO enrichment analysis revealed that BBCT was mainly involved in the neuropeptide signaling pathway in biological processes, extracellular regions in cellular components, and RNA methyltransferase activity in molecular functions. Notably, the neuropeptide signaling pathway includes several genes involved in the neuroactive ligand-receptor interaction pathway, such as *Cysltr1*, *2*, *Grp*, *Hcrtr1*, *2*, *Npb*, *Ptgdr*, and *Qrfp*. Additional biological processes in which BBCT was found to be involved included apoptotic cell death involving TRAF1, positive regulation of cell proliferation, cell differentiation, and angiogenesis.

Our study has several limitations. The hippocampus plays a crucial role in memory and cognition, and hippocampal lesions are important in the pathogenesis of VD. Moreover, impaired white matter integrity can contribute to cognitive impairment induced by chronic cerebral hypoperfusion. However, we did not observe the activation of glial cells in the mice hippocampus and the white matter lesions, which should be investigated in future studies 25. Second, the activation of microglia and astrocytes plays a crucial role in ischemic injury; however, the role of these glial cells remains unclear, and we did not investigate the inflammatory cytokines and ROS in serum or brain tissue. Microglia and astrocytes have two opposite phenotypes: M1 and M2 for microglia, and A1 and A2 for astrocytes. M1 microglia and A1 astrocytes cause neuroinflammation; however, M2 microglia and A2 astrocytes exert neuroprotective effects by releasing anti-inflammatory factors and supporting neuronal repair 26,27. Lu et al. 50 confirmed that Shaoyao-Gancao decoction decreased M1 microglia and increased M2 microglia in MCAO model rats. Future studies should characterize the effect of BBCT on the polarization of glial cells and identify the effects of BBCT on ROS or inflammatory cytokines. Third, we did not quantitively analyze the main proteins that play a key role in many PPI networks. In future studies, the Cytohubba plugin in Cytoscape software 51 should also be used to analyze the major target proteins constituting the PPI network for multifaceted analysis. Furthermore, quantitative analyses of the effects of BBCT on changes in the expression of major target proteins and proteins that play a major role in major mechanisms should be performed. Fourth, our study did not include an active control group using anti-dementia drugs. Future studies comparing BBCT with other drugs for VD, such as donepezil, are needed.

## Conclusions

BBCT ameliorated BCAS-induced cognitive impairment and attenuated activation of microglia and astrocyte induced by BCAS. Moreover, BBCT restored 79.8% and 76.7% of the genes' expressions were up-regulated and down-regulated by BCAS, respectively. BBCT may have a neuroprotective effect against BCAS-induced cerebral ischemic injury in mice, possibly by regulating the expression of CDC20, EGF, TRAF1, and key proteins in neuroactive ligand-receptor interaction and neuropeptide signaling pathways. Our results provide a foundation for future studies on the underlying mechanisms of BBCT in patients with VD.

## Supplementary Material

Supplementary figures and tables.

## Funding

This work was supported by the National Research Foundation of Korea (NRF) grant funded by the Korea government (MSIT) (No. 2021R1F1A1055614).

## Author Contributions

Conceptualization, Sang-Ho Kim and Suin Cho; methodology, Suin Cho; software, Suin Cho; formal analysis, Suin Cho; investigation, Da-Woon Kim and Suin Cho; resources, Suin Cho; data curation, Da-Woon Kim and Suin Cho; writing-original draft preparation, Da-Woon Kim and Jung-Hwa Lim; writing-review and editing, Sang-Ho Kim; visualization, Suin Cho; supervision, Sang-Ho Kim; project administration, Sang-Ho Kim and Jung-Hwa Lim; funding acquisition, Jung-Hwa Lim. All authors have read and agreed to the published version of the manuscript.

## Figures and Tables

**Table 1:** Composition of Banhabaekchulcheonma-Tang (BBCT) used in this study

Scientific name (Herbal name)	Chinese herbal name	Amount (g)
*Pinellia ternata* (Pinelliae Tuber)	半夏 (banxia)	12.00
*Citrus unshiu* (Citri Unshius Pericarpium)	陳皮 (chenpí)	12.00
*Hordeum vulgare* var.* hexastichon* (Hordei Fructus Germinatus)	麥芽炒 (maiyachao)	12.00
*Zingiber officinale* (Zingiberis Rhizoma Recens)	生薑 (shengjiang)	12.00
*Atractylodes japonica* (Atractylodis Rhizoma Alba)	白朮 (baizhu)	8.00
*Triticum aestivum L.* (Massa Medicata Fermentata)	神麯炒 (shenquchao)	8.00
*Atractylodes lancea* (Atractylodis Rhizoma)	蒼朮 (cangzhu)	8.00
*Panax ginseng* (Ginseng Radix)	人蔘 (renshen)	4.00
*Astragalus membranaceus* (Astragali Radix)	黃芪 (huangqi)	4.00
*Poria cocos* (Poria Sclerotium)	白茯苓 (baifuling)	4.00
*Gastrodia elata* (Gastrodiae Rhizoma)	天麻 (tianma)	4.00
*Alisma orientale* (Alismatis Rhizoma)	澤瀉 (zexie)	4.00
*Zingiber officinale* (Zingiberis Rhizoma)	乾薑 (ganjiang)	2.00
*Phellodendron amurense* (Phellodendri Cortex)	黃柏 (huangbo)	2.00

**Table 2:** Identification of key pathways affected by BBCT administration identified by KEGG pathway analysis

Term	Genes count	*P*-value
Neuroactive ligand-receptor interaction	15	1.30E-04
Cytokine-cytokine receptor interaction	10	6.50E-03
Starch and sucrose metabolism	4	6.90E-03
Salivary secretion	5	1.70E-02
Carbohydrate digestion and absorption	4	1.80E-02
Pancreatic secretion	5	4.30E-02
Prolactin signaling pathway	4	5.40E-02
Breast cancer	5	9.00E-02
IL-17 signaling pathway	4	9.30E-02

Significantly enriched KEGG pathways were listed based on *P* < 0.05.

## Abbreviations

AChEIs: Acetylcholinesterase inhibitors
ANOVA: Analysis of variance
APC: Anaphase-promoting complex
Aβ: Amyloid-β
BBCT: Banhabaekchulcheonma-Tang
BCAS: Bilateral carotid artery stenosis
CDC20: Cell division cycle protein 20
CYSLTR: cysteinyl leukotriene receptor
EGF: Epidermal growth factor
GO: Gene Ontology
GRP: Gastrin-releasing peptide
HTR: 5-hydroxytryptamine receptor
IL-17: Interleukin-17
KEGG: Kyoto Encyclopedia of Genes and Genomes
MCAO: Middle cerebral artery occlusion
nAChR: Nicotinic acetylcholine receptor
NF-κB: Nuclear factor kappa light chain enhancer of activated B cells
NORT: Novel object recognition test
Nrf2: Nuclear factor erythroid-2-related factor 2
PBS: Phosphate-buffered saline
PPI: Protein-protein interaction
ROS: Reactive oxygen species
STRING: Search Tool for the Retrieval of Interacting Genes/Proteins
TNF-α: Tumor necrosis factor-α
TRAF1: Tumor necrosis factor receptor-associated factor 1
VD: Vascular dementia
