# Supplementary material for: Effects of Banhabaekchulcheonma-Tang on Brain Injury and Cognitive Function Impairment Caused by Bilateral Common Carotid Artery Stenosis in a Mouse Model
Source: Int J Med Sci. 2024 Jan 21;21(4):644–55. doi: 10.7150/ijms.90167 (PMC10920841; doi:10.7150/ijms.90167)
Supplement: Supplementary file 1 — Supplementary figures and tables. [file ijmsv21p0644s1.pdf]

Table S1. Gene Ontology term analysis of biological processes

Term	Gene count	*P*-Value
Neuropeptide signaling pathway	7	3.90E-04
Cilium organization	4	3.30E-03
Signal transduction	26	6.40E-03
Sleep	3	7.00E-03
Positive regulation of macrophage derived foam cell differentiation	3	8.30E-03
Spermatogenesis	13	9.80E-03
Grooming behavior	3	1.40E-02
Lens morphogenesis in camera-type eye	3	1.40E-02
Maternal behavior	3	1.40E-02
Cell differentiation	20	1.40E-02
Multicellular organism development	21	1.40E-02
Positive regulation of cell proliferation	14	2.10E-02
Calcium ion transport	6	2.20E-02
Immune system process	12	2.70E-02
Response to external biotic stimulus	2	3.20E-02
Negative regulation of hair follicle development	2	3.20E-02
Female pregnancy	5	3.40E-02
Positive regulation of cytokine production involved in inflammatory response	3	3.60E-02
Apoptotic signaling pathway	4	3.70E-02
Maternal aggressive behavior	2	4.20E-02

Cellular response to hormone stimulus	3	4.30E-02
Sperm axoneme assembly	3	4.30E-02
Nervous system development	10	4.70E-02
Positive regulation of blood pressure	3	4.90E-02
Apoptotic process	13	4.90E-02
Positive regulation of hyaluronan biosynthetic process	2	5.30E-02
T cell homeostasis	3	6.00E-02
Positive regulation of autophagy	4	6.00E-02
Sperm ejaculation	2	6.30E-02
Saliva secretion	2	6.30E-02
Positive regulation of I-kappaB kinase/NF-kappaB signaling	5	7.20E-02
Negative regulation of neuron projection development	4	7.30E-02
Protein localization to chromosome	2	7.30E-02
Fatty acid metabolic process	6	7.40E-02
Regulation of G-protein coupled receptor protein signaling pathway	3	8.10E-02
Negative regulation by host of viral genome replication	2	8.30E-02
Angiogenesis	7	8.60E-02
Circadian rhythm	4	8.60E-02
Cilium assembly	6	9.00E-02
Cytokine-mediated signaling pathway	5	9.10E-02
Positive regulation of NF-kappaB transcription	5	9.20E-02

factor activity		
Negative regulation of glial cell apoptotic process	2	9.30E-02
Positive regulation of lipid catabolic process	2	9.30E-02

Significantly enriched GO terms were listed based on $P < 0.05$.

Table S2. Gene Ontology term analysis of cellular components

**Term**	**Gene count**	***P*-Value**
Extracellular region	36	4.80E-04
Membrane	90	5.50E-03
Cilium	11	9.20E-03
Receptor complex	8	1.00E-02
Ciliary basal body	7	1.20E-02
Cell projection	20	4.00E-02
Motile cilium	6	4.10E-02
Neuron projection	12	4.50E-02
Ripoptosome	2	5.10E-02
Cytoskeleton	22	5.50E-02
Muscle tendon junction	2	6.10E-02
Male germ cell nucleus	4	6.10E-02
Centrosome	11	6.40E-02
Cytoplasm	89	7.10E-02
Endoplasmic reticulum membrane	15	8.10E-02
Centriole	5	8.30E-02

Significantly enriched GO terms were listed based on $P < 0.05$.

Table S3. Gene Ontology term analysis of molecular function

Term	Gene count	*P*-Value
RNA methyltransferase activity	3	7.50E-03
Monosaccharide binding	3	1.70E-02
Neuropeptide hormone activity	3	3.80E-02
Monooxygenase activity	5	4.00E-02
Alpha-amylase activity	2	4.30E-02
Tumor necrosis factor receptor binding	3	5.40E-02
G-protein coupled receptor binding	4	5.50E-02
Iron ion binding	6	5.90E-02
Ubiquitin protein ligase binding	8	6.40E-02
Oxidoreductase activity, acting on paired donors, with incorporation or reduction of molecular oxygen	4	7.20E-02
Microtubule binding	7	7.20E-02
Peptide hormone binding	3	8.20E-02
O-methyltransferase activity	2	8.50E-02
Calcium-dependent protein binding	4	9.00E-02
tRNA methyltransferase activity	2	9.50E-02

Significantly enriched GO terms were listed based on $P < 0.05$.

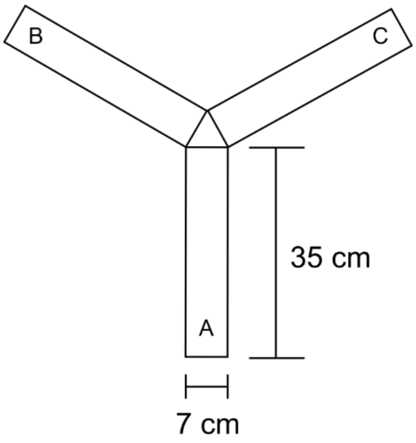

Figure S1. Diagrammatic representation of the Y-maze. Each arm is positioned at an equal angle of 120 degrees and is 35 cm long, 7 cm wide, and 40 cm high.

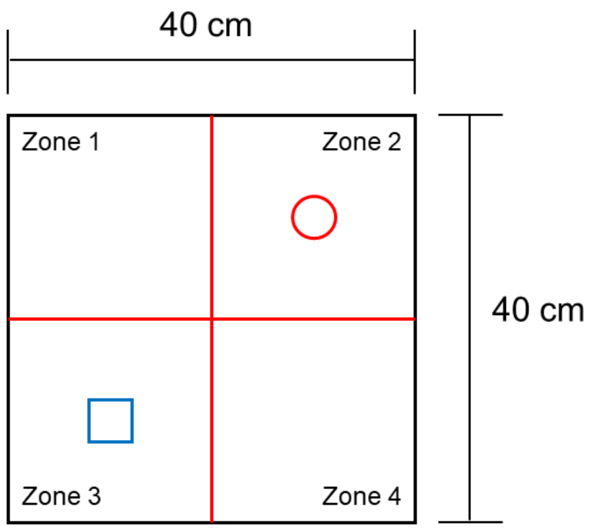

Figure S2. Field box specifications for the novel object recognition test. A square chamber with a width, length, and height of 40 cm was used. After the acquisition session with the same object in Zones 2 and 3, the object in Zone 3 was substituted by a new object.

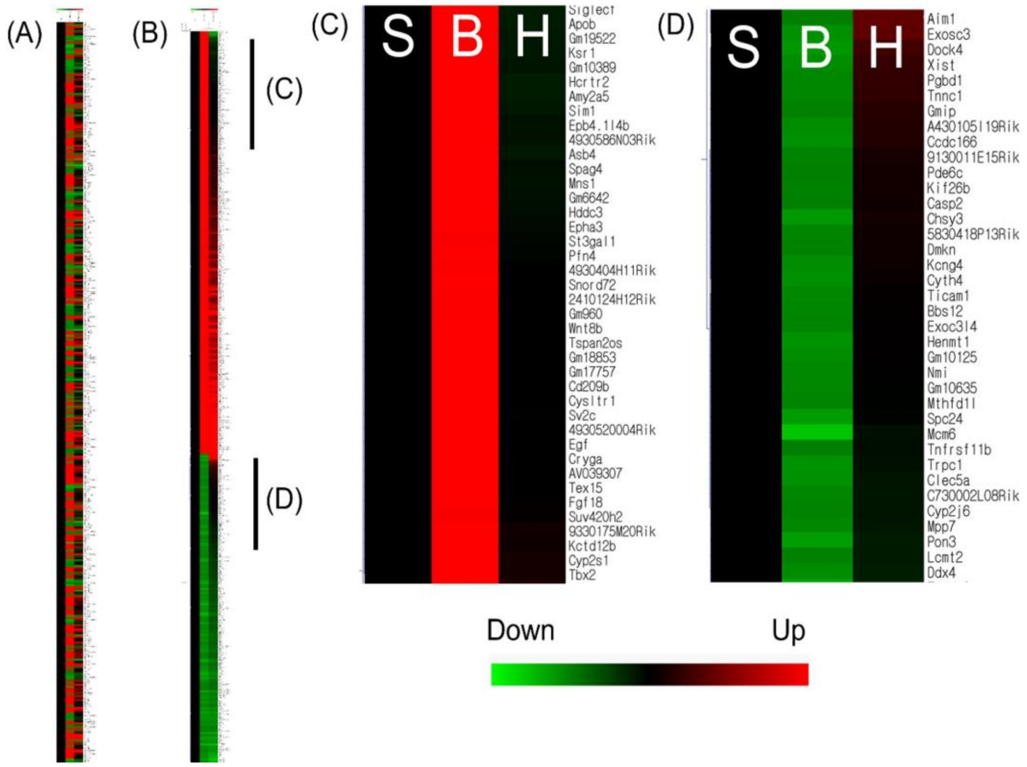

Figure S3. Effects of H-BBCT on gene expressions in the brain cortex of BCAS model mice. (A) Heat map illustrating the expression profiles of the differentially expressed genes. (B) Genes with 2-fold down-regulation or up-regulation compared to the sham-operated group were listed separately. (C) Genes up-regulated due to BCAS surgery restored by H-BBCT. (D) Genes down-regulated due to BCAS surgery restored by H-BBCT. Black represents the gene expression levels from the sham-operated group used as a reference. Green represents genes with 2-fold down-regulation compared to the sham-operated group; red represents genes with 2-fold up-regulation compared to the sham-operated group.

S: Sham-operated and distilled water-administered group, B: BCAS-operated and distilled water-administered group, H: BCAS-operated and H-BBCT-administered group (H-BBCT group)

BBCT, Banhabaekchulcheonma-Tang; BCAS, bilateral carotid artery stenosis

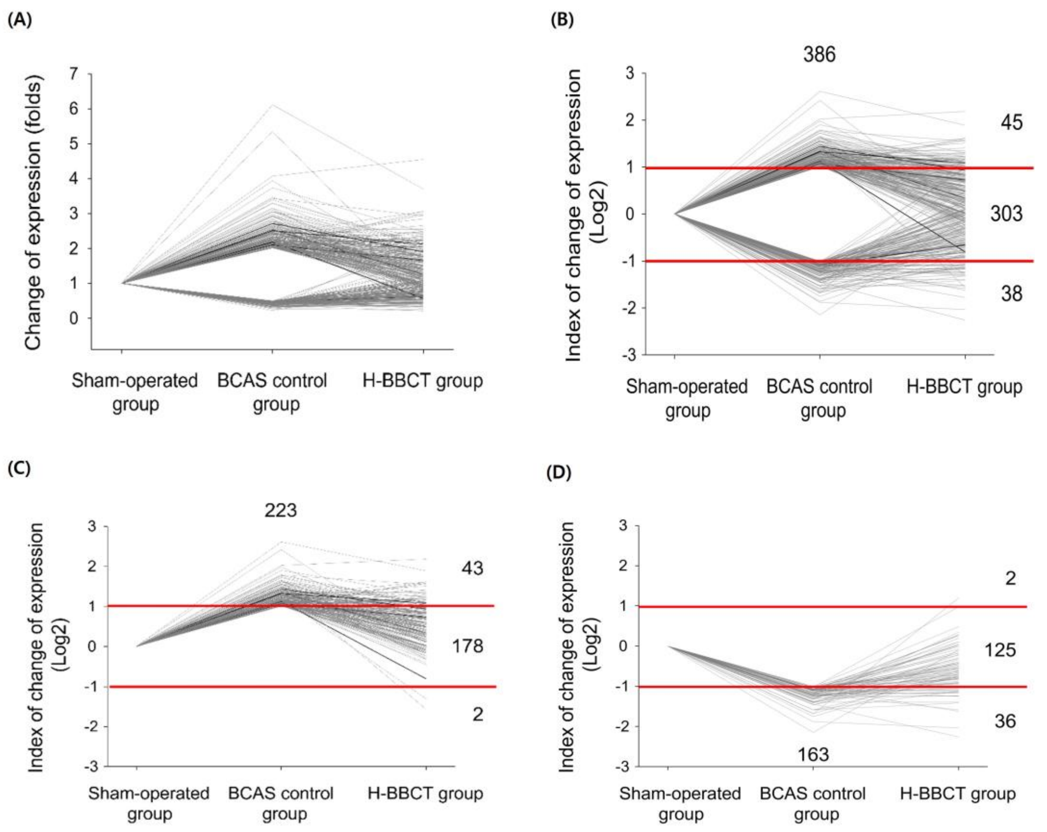

Figure S4. Gene expression changes in BCAS model mice are presented as line plots. For each gene with differential expression levels compared to the sham-operated group, the raw fold values (A) were converted to log values (B), and the number of up-regulated (C), down-regulated (D), and restored genes were presented (C, D).

BCAS, bilateral carotid artery stenosis
